# COVID-19 vaccine equity: a retrospective population-based cohort study examining primary series and first booster coverage among persons with a history of immigration and other residents of Ontario, Canada

**DOI:** 10.3389/fpubh.2023.1232507

**Published:** 2023-09-08

**Authors:** Susitha Wanigaratne, Hong Lu, Sima Gandhi, Janavi Shetty, Therese A. Stukel, Pierre-Philippe Piché-Renaud, Julia Brandenberger, Samiya Abdi, Astrid Guttmann

**Affiliations:** ^1^Edwin S.H. Leong Center for Healthy Children, University of Toronto, Toronto, ON, Canada; ^2^ICES, Toronto, ON, Canada; ^3^Institute of Health Policy, Management and Evaluation, University of Toronto, Toronto, ON, Canada; ^4^Dalla Lana School of Public Health, University of Toronto, Toronto, ON, Canada; ^5^Division of Infectious Diseases, The Hospital for Sick Children, Toronto, ON, Canada; ^6^Division of Pediatric Emergency Medicine, Hospital for Sick Children, Toronto, ON, Canada; ^7^Pediatric Emergency Department, University Hospital of Bern, Bern, Switzerland; ^8^Public Health Ontario, Toronto, ON, Canada; ^9^Division of Pediatric Medicine, Department of Pediatrics, Hospital for Sick Children, Toronto, ON, Canada

**Keywords:** COVID-19 vaccine, immigrants, refugees, public health, Ontario (Canada), social determinants of health

## Abstract

**Introduction:**

Immigrants were disproportionately impacted by COVID-19 and experience unique vaccination barriers. In Canada (37 million people), 23% of the population is foreign-born. Immigrants constitute 60% of the country’s racialized (non-white) population and over half of immigrants reside in Ontario, the country’s most populous province. Ontario had several strategies aimed at improving vaccine equity including geographic targeting of vaccine supply and clinics, as well as numerous community-led efforts. Our objectives were to (1) compare primary series vaccine coverage after it was widely available, and first booster coverage 6 months after its availability, between immigrants and other Ontario residents and (2) identify subgroups experiencing low coverage.

**Materials and methods:**

Using linked immigration and health administrative data, we conducted a retrospective population-based cohort study including all community-dwelling adults in Ontario, Canada as of January 1, 2021. We compared primary series (two-dose) vaccine coverage by September 2021, and first booster (three-dose) coverage by March 2022 among immigrants and other Ontarians, and across sociodemographic and immigration characteristics. We used multivariable log-binomial regression to estimate adjusted risk ratios (aRR).

**Results:**

Of 11,844,221 adults, 22% were immigrants. By September 2021, 72.6% of immigrants received two doses (vs. 76.4%, other Ontarians) and by March 2022 46.1% received three doses (vs. 58.2%). Across characteristics, two-dose coverage was similar or slightly lower, while three-dose coverage was much lower, among immigrants compared to other Ontarians. Across neighborhood SARS-CoV-2 risk deciles, differences in two-dose coverage were smaller in higher risk deciles and larger in the lower risk deciles; with larger differences across all deciles for three-dose coverage. Compared to other Ontarians, immigrants from Central Africa had the lowest two-dose (aRR = 0.60 [95% CI 0.58–0.61]) and three-dose coverage (aRR = 0.36 [95% CI 0.34–0.37]) followed by Eastern Europeans and Caribbeans, while Southeast Asians were more likely to receive both doses. Compared to economic immigrants, resettled refugees and successful asylum-claimants had the lowest three-dose coverage (aRR = 0.68 [95% CI 0.68–0.68] and aRR = 0.78 [95% CI 0.77–0.78], respectively).

**Conclusion:**

Two dose coverage was more equitable than 3. Differences by immigrant region of birth were substantial. Community-engaged approaches should be re-invigorated to close gaps and promote the bivalent booster.

## Introduction

Vaccine equity means that everyone should have fair and just access to vaccines. Inequities are not merely differences between groups; they disproportionately impact those most disadvantaged by social structures, are avoidable, and can be mitigated through targeted social policies (1). While inequities by race/ethnicity in SARS-CoV-2 infection, vaccination, and severe outcomes occurred in many countries (2, 3), studies of COVID-19 vaccination within and between im/migrant populations are limited (4–10), despite racialized and ethnic communities often consisting of substantial numbers of immigrants experiencing unique barriers to vaccination (4, 11–15). A recent systematic review among migrants in Europe (4) identified several determinants of under-vaccination including geographic origin, recent migration, being a refugee or asylum-seeker, or not accessing health care in the past 12 months.

As of 2021, 23% of Canada’s population was foreign-born (~8.4 million people) with 70% of immigrants belonging to a race or ethnicity other than White (16). Each year between 300,000 and 400,000 immigrants become permanent residents with the majority (~60%) arriving as economic immigrants, including the caregiver class, who meet specific criteria for pre-existing medical conditions, education, knowledge of Canada’s official languages, and ability to contribute to the economy. The remainder arrive as sponsored family (~30%), resettled refugees (~5%), or asylum-seekers (~5%) and are generally not selected on the same criteria. Over half of all immigrants to Canada, reside in Ontario, Canada’s most populous province (15.5 million). Immigrants and refugees (17) and racialized Canadians have been disproportionately impacted by SARS-CoV-2 infection (18) and COVID-19 related severe outcomes (19), understood to be the result of structural and social conditions (e.g., systemic racism, discrimination, and high-risk jobs). In addition to other public health protections (e.g., masking), COVID-19 vaccinations are critical to minimize infections and severity.

Equitable distribution has been a concern in Ontario (20) since the COVID-19 vaccine rollout began. Vaccines were available at no cost for all individuals in Ontario, and proof of identity with name and date of birth including Ontario provincial health insurance card or foreign and expired documents were accepted (21). In April 2021, a few weeks ahead of availability to the wider adult population, the Ontario government allocated additional vaccines to 30% (*n* = 114) of neighborhoods which had historically high rates of COVID-19 related deaths and hospitalizations; 50% of available vaccines were allocated to these neighborhoods for 2 weeks in May 2021 with the age of eligibility lower than in other neighborhoods (22). In addition, public health and their hospital and community partners used geographically disaggregated vaccination data to identify eligibility for and locations of appointment-based mass vaccination and pop-up and hospital clinics (20). Participating pharmacies, with walk-in appointments and mobile and community-led and culturally safe clinics (23–25) were utilized later in the vaccine campaign. Importantly, these community-led vaccination efforts aimed to reduce inequities and achieve high primary series coverage. While public health maintained a focus on equity, booster uptake was negatively associated with neighborhood marginalization and diversity (26).

The objectives of this study were to: (i) compare primary series (two-dose) vaccine coverage, measured after the series was vigorously promoted, and first booster (three-dose) coverage, measured 6 months after its widespread availability, between immigrants residing in the province of Ontario, Canada, and other Ontarians and across sociodemographic, primary care, and immigration characteristics; and (ii) model the association between vaccination status and these characteristics among all Ontarians and immigrants separately. We hypothesized that: (i) overall and within strata of characteristics, immigrants would be less likely to receive both second and third doses compared to other Ontarians; (ii) overall and within strata of characteristics, differences in vaccination coverage between immigrants and other Ontarians would be smaller for two than three doses, (iii) those residing in higher neighborhood SARS-CoV-2 risk deciles would have the highest primary series coverage, and (iv) among immigrants, region of birth, duration of residence, refugee status, and primary care enrollment would be important determinants of vaccination. To the best of our knowledge, this is one of six (5–9) published studies examining COVID-19 vaccination among immigrants in high-income countries, and the first to report on the primary series and first booster using a total population-based denominator.

## Methods

### Study design and cohort

We conducted a retrospective population-based cohort study using administrative databases in Ontario, Canada available at ICES (formerly Institute for Clinical Evaluative Sciences). ICES is an independent, non-profit research institute whose legal status under Ontario’s health information privacy law allows it to collect and analyze health care and demographic data, without consent, for health system evaluation and improvement. Immigration and healthcare databases were linked using unique encoded identifiers, derived from provincial health card numbers and analyzed at ICES. For privacy and security purposes, in addition to physical safeguards at ICES (e.g., complex passwords, encryption), only co-author HL accessed de-identified datasets to conduct analyses and generate output, suppressing cell counts of ≤5 where appropriate. All community-dwelling Ontario residents ≥18 years eligible for publicly funded health insurance as of January 1, 2021 were included and followed for vaccine coverage at two time points (see primary exposures). Since the immigration database included immigrants arriving between January 1, 1985 and September 30, 2020, we excluded anyone who registered for healthcare after September 30, 2020 since these may be immigrants who could not be identified. Cohort members who left Ontario or died during the follow-up were retained for all analyses. To help ensure individuals were residing in Ontario as of the study start date, we excluded Ontario residents who were < 65 years old on January 1, 2021 who did not use the health system within the previous 9 years and those who were ≥ 65 who did not use the health system within the previous 3 years (27). This single-payer system provides access to most medically necessary healthcare services including primary healthcare (e.g., annual physical exam), emergency room visits, hospital admissions, lab testing etc. (28). We followed relevant reporting guidelines (29).

### Data sources

Several data sources were linked to identify the population of interest and operationalize outcomes, exposures and covariates. Details on these databases and the associated variables are described in Supplementary Tables 1, 2.

### Primary exposures

The two time points at which vaccination was assessed were the main exposures. The first (September 13, 2021), was when the provincial mandatory vaccination program was put in place and proof of two-dose vaccination was needed for adults to enter high-risk indoor public settings. In anticipation of the program, the primary series was promoted extensively. The second time point, March 13, 2022, was approximately 6 months after the first booster became widely available and coincided with the end of the mandatory program. See Supplementary Table 3 for timelines.

### Secondary exposure

In descriptive analyses, persons with a history of immigration to Canada (specifically arriving in the province of Ontario between 1985 and 2020, henceforth referred to as “immigrants”) were compared to “other Ontarians.” This included all adults not identified as immigrants using the immigration database. Other Ontarians were conceptualized as experiencing the fewest vaccination barriers on average. In multivariable models, immigrant region of birth was the exposure of interest and conceptualized as a proxy for (i) identifying racialized immigrants, some of whom experience barriers to vaccine information and access stemming from social exclusion (13) and (ii) cultural, religious, and/or linguistic barriers to vaccination, which may be sustained after migration or spread through transnational networks (30, 31) and for which tailored outreach may be possible. In models including all Ontarians, the reference group was “other Ontarians.” In immigrant-focused models, South-east Asians were the reference because they had the highest vaccination coverage.

### Outcome measures

In descriptive analyses, at the first time point vaccine coverage was described as being unvaccinated, had one dose, and completed the primary series (henceforth referred to as “two-dose”). At the second time point, first booster (henceforth referred to as “three-dose”) coverage was also examined. In multivariable regression we modeled two-dose coverage at the first time point and three-dose coverage at the second time.

### Covariates

Sociodemographic variables included sex and age group. Primary care affiliation was conceptualized as an indicator of access to trusted vaccine counseling and a source of information on evolving vaccine eligibility and access (Supplementary Table 3). Neighborhood income quintile was considered a contextual measure of socio-economic status. Neighborhood SARS-CoV-2 risk deciles were based on the cumulative incidence of SARS-CoV-2 as of March 28, 2021 in geographic regions (~8,000 households) which were ranked from highest (decile 1) to lowest (decile 10) risk, such that each decile had an equal number of neighborhoods. In April 2021, these risk deciles along with neighborhood COVID-19 related hospitalizations and deaths were used to more equitably distribute vaccines (20); specifically 30% of high risk neighborhoods were allocated 50% of available vaccines for 2 weeks in May 2021 with the age of eligibility lower than in other neighborhoods (22). Immigration category specifies an immigrant’s pathway to permanent residency; these pathways differ according to how an immigrant is selected which include pre-existing medical conditions, education, and official language ability at arrival which further relate to employment and social conditions after arrival. Immigrant duration of residence was conceptualized as a proxy for increasing understanding and/or resources needed to navigate Canadian systems and services.

### Statistical analysis

#### Descriptive analyses

We used standardized differences (SDs) (32) to compare the distribution of sociodemographic and primary care model characteristics between immigrants and other Ontarians, where groups are considered balanced when SD ≤ 0.1. We compared two- and three-dose coverage between immigrants and other Ontarians by calculating the difference in coverage, overall, and within strata. Statistical tests (e.g., χ^2^) were not conducted given the use of population data removes the need for inference and that our large population sizes would lead to statistically significant differences (*p* < 0.05). Instead, we considered differences in coverage greater than 5% to have public health significance.

#### Multivariable modeling

We aimed to identify the most important characteristics associated with two- and three-dose coverage using multivariable log-binomial regression. All Ontarian models included terms for age, sex, primary care enrollment model, neighborhood risk decile with immigrant region of birth as our main interest due to the diversity of immigrant communities in Ontario, and the consistent association of under-vaccination with geographic origin (4). Age and sex were included due to their standard inclusion. Neighborhood risk decile was included due to their use by public health in vaccine allocation early in the rollout. Due to the high correlation between neighborhood income and neighborhood risk decile, the former was not included. Immigrant-focused models additionally included immigration category, as these potentially relate to challenging circumstances (e.g., refugees) and employment-related motivation for vaccination (e.g., caregivers), and duration of residence, as a proxy for increasing ability to navigate healthcare. Inclusion of these immigration variables were also supported by a recent review (4). While some of these variables may mediate the effect of other variables on vaccine coverage (e.g., immigrant region of birth →immigration category → vaccination), we aimed to identify associations independent of potential mediation. Adjusted risk ratios (aRR) and 95% confidence intervals (CI) were reported. Significant differences between two and three-dose coverage were identified with non-overlapping 95% CIs on forest plots.

### Ethics approval

ICES is a prescribed entity under Ontario’s Personal Health Information Protection Act (PHIPA). The use of these project data is authorized under section 45 of PHIPA and does not require review by a Research Ethics Board.

## Results

### Baseline characteristics

After exclusions (see Supplementary Figure 1 for cohort selection flow chart), of all adult Ontarians (*N* = 11,844,221), 22% (*N* = 2,565,374) were immigrants (Table 1). Compared to other Ontarians, immigrants were more likely to be <65 years old and to live in neighborhoods in the lowest income quintile, and less likely to live neighborhoods in the highest income quintile. Immigrants were two to three times more likely to live in neighborhoods in the highest three SARS-CoV-2 risk deciles.

**Table 1:** Socio-demographic, primary care, and immigration characteristics for persons with a history of immigration and other Ontario residents as of January 1, 2021, *n* (column %).

		Persons with a history of immigration	Other Ontario residents	SD^a^
				Immigrants vs. other ON
	Total population	2,565,374	9,278,847	
Sex	Female	1,336,536 (52.1%)	4,717,340 (50.8%)	0.03
	Male	1,228,838 (47.9%)	4,561,507 (49.2%)	0.03
Age	18–44	1,250,684 (48.8%)	4,037,699 (43.5%)	0.11
	45–64	978,382 (38.1%)	3,030,519 (32.7%)	0.11
	65+	336,308 (13.1%)	2,210,629 (23.8%)	0.28
Neighborhood income	1 (lowest)	632,430 (24.7%)	1,724,229 (18.6%)	0.15
	2,3,4	1,568,357 (61.1%)	5,531,407 (59.6%)	0.03
	5 (highest)	364,587 (14.2%)	2,023,211 (21.8%)	0.2
Primary care	Primary care enrollment model	2,034,680 (79.3%)	7,701,546 (83.0%)	0.09
	Other	334,691 (13.0%)	899,546 (9.7%)	0.01
	No primary care	196,003 (7.6%)	677,755 (7.3%)	0.11
Neighborhood SARS-CoV-2 risk deciles	1(highest risk)	524,393 (20.5%)	627,751 (6.8%)	0.41
	2	472,273 (18.5%)	708,680 (7.7%)	0.32
	3	369,842 (14.5%)	776,930 (8.4%)	0.19
	4	273,843 (10.7%)	910,473 (9.8%)	0.03
	5	232,810 (9.1%)	947,884 (10.2%)	0.04
	6	210,155 (8.2%)	1,024,445 (11.1%)	0.1
	7	212,263 (8.3%)	946,372 (10.2%)	0.07
	8	155,801 (6.1%)	996,858 (10.8%)	0.17
	9	67,455 (2.6%)	1,144,006 (12.4%)	0.38
	10 (lowest risk)	39,750 (1.6%)	1,168,317 (12.6%)	0.44
Immigration category	Sponsored family	891,323 (34.7%)	n/a	
	Economic caregiver	102,717 (4.0%)	n/a	
	Economic other	1,108,348 (43.2%)	n/a	
	Resettled refugee	196,668 (7.7%)	n/a	
	Protected persons	223,268 (8.7%)	n/a	
	Other immigrants	43,050 (1.7%)	n/a	
Region of birth	Central Africa	12,363 (0.5%)	n/a	
	Western Africa	48,837 (1.9%)	n/a	
	East Africa	81,640 (3.2%)	n/a	
	Southern Africa	12,382 (0.5%)	n/a	
	Middle East	237,903 (9.3%)	n/a	
	North Africa	44,646 (1.7%)	n/a	
	Central America	51,764 (2.0%)	n/a	
	South America	127,400 (5.0%)	n/a	
	Caribbean	152,552 (5.9%)	n/a	
	North America	49,649 (1.9%)	n/a	
	East Asia	365,918 (14.3%)	n/a	
	Australasia & Oceania	7,685 (0.3%)	n/a	
	Southeast Asia	281,303 (11.0%)	n/a	
	South Asia	676,005 (26.4%)	n/a	
	Eastern Europe	230,523 (9.0%)	n/a	
	Other Europe	184,757 (7.2%)	n/a	
	Not stated	47 (0.0%)	n/a	
Duration of residence	Under 5 years	396,811 (15.5%)	n/a	
	5–9 years	359,885 (14.0%)	n/a	
	10–19 years	841,828 (32.8%)	n/a	
	20+ years	966,850 (37.7%)	n/a	

a SD, standardized differences ≤ 0.1 indicate characteristics are balanced between immigrants and other Ontario residents.

### Describing vaccination status: comparing immigrants to other Ontarians within strata

By September 2021, 72.6% of immigrants received two doses compared to 76.4% for other Ontarians (Table 2A). By March 2022, 46.1% of immigrants received three doses (16.3% had zero doses) compared to 58.2% for other Ontarians (13.8% had zero doses; Table 2B). Overall, there were few large differences in two-dose coverage across strata comparing immigrants to other Ontarians; however, for three-dose coverage differences were larger and more numerous. Immigrants living in the highest income quintile had lower two-dose coverage, while immigrants in all income quintiles had lower three dose coverage and these differences increased with neighborhood income. Across neighborhood risk deciles, differences in two-dose coverage between immigrants and other Ontarians were smaller in the higher risk deciles and larger in the lower risk deciles. For three-dose coverage, differences were larger across all risk deciles. Among those with no primary care access in the previous 2 years, immigrants had lower two-dose coverage than other Ontarians; but regardless of primary care model, immigrants had lower three-dose coverage.

**Table 2A:** Proportion (row % unless otherwise indicated) of persons with a history of immigration to Ontario (1985-2020) and other Ontarians by COVID-19 vaccination status and difference in two-dose vaccination coverage as of September 13, 2021 across sociodemographic, primary care and immigration characteristics.

		Persons with a history of immigration (row %)			Other Ontarians (row %)			Immigrants vs. other Ontarians
		% 2 doses	% 1 dose	% 0 doses	% 2 doses	% 1 dose	% 0 doses	Difference in % 2 doses^a^
	Total Population	72.6	5.3	22.1	76.4	4.8	18.8	−3.8
Sex	Female	73.4	5.1	21.5	78.6	4.3	17.1	−5.2
	Male	71.8	5.4	22.8	74.2	5.4	20.5	−2.4
Age	18-44	68.8	6.9	24.4	66.4	7.2	26.4	2.4
	45-64	76.7	4.0	19.3	80.8	3.7	15.5	−4.1
	65+	75.2	2.9	21.9	88.8	2.0	9.2	−13.6
Neighborhood Income	1 (lowest)	68.8	6.4	24.8	69.0	6.4	24.7	−0.2
	2,3,4	73.9	5.1	21.1	76.8	4.8	18.4	−2.9
	5 (highest)	73.8	4.1	22.0	81.9	3.6	14.6	−8.1
Primary Care	Primary Care Enrollment model	75.6	5.0	19.4	79.3	4.5	16.2	−3.7
	Other	70.0	6.8	23.3	69.2	6.6	24.1	0.8
	No primary care	46.2	5.4	48.4	53.4	6.4	40.2	−7.2
Neighborhood SARS-CoV-2 Risk Deciles	1(highest risk)	74.0	6.0	20.0	71.7	6.1	22.2	2.3
	2	75.2	4.8	19.9	76.3	4.7	19.1	−1.1
	3	71.6	5.3	23.1	76.2	4.7	19.1	−4.6
	4	70.0	5.7	24.2	75.0	5.1	19.9	−5.0
	5	72.6	5.0	22.5	76.9	4.6	18.5	−4.3
	6	70.9	5.1	24.0	77.2	4.7	18.2	−6.3
	7	71.7	4.6	23.7	78.8	4.1	17.1	−7.1
	8	72.4	4.8	22.8	78.6	4.3	17.1	−6.2
	9	69.7	5.3	25.0	77.1	4.8	18.1	−7.4
	10 (lowest risk)	70.8	5.0	24.2	75.0	5.4	19.6	−4.2
Immigration Category	Sponsored Family	70.7	5.5	23.7				−5.7
	Economic Caregiver	86.0	4.4	9.5				9.6
	Economic Other	75.6	3.9	20.5				0.8
	Resettled Refugee	63.7	8.8	27.5				−12.7
	Protected persons	67.7	7.9	24.4				−8.7
	Other immigrants	68.6	6.8	24.6				−7.8
Region of Birth	Central Africa	42.2	3.5	44.4				−34.2
	Western Africa	63.4	9.5	27.1				−13.0
	East Africa	63.6	9.5	27.0				−12.8
	Southern Africa	77.8	3.5	18.7				1.4
	Middle East	68.2	7.2	24.6				−8.2
	North Africa	66.4	6.8	26.9				−10.0
	Central America	69.2	6.1	24.8				−7.2
	South America	75.4	5.1	19.5				−1.0
	Caribbean	55.8	7.6	36.6				−20.6
	North America	64.2	5.0	30.8				−12.2
	East Asia	78.8	2.7	18.5				2.4
	Australasia & Oceania	71-76 ^b^	2-6 ^b^	22-27 ^b^				−0.4 to −5.4 ^b^
	Southeast Asia	86.3	3.6	10.1				9.9
	South Asia	80.6	4.9	14.5				4.2
	Eastern Europe	51.5	5.8	42.7				−24.9
	Other Europe	67.3	5.4	27.3				−9.1
	Not Stated	^b^	^b^	^b^				b
Duration of Residence	Under 5 years	71.7	7.0	21.3				−4.7
	5 to 9 years	69.5	5.7	24.7				−6.9
	10 to 19 years	72.8	5.1	22.1				−3.6
	20+ years	74.0	4.5	21.5				−2.4

a Difference in % for immigration category, region of birth and duration of residence are vs. % 2 doses for other Ontarians overall (76.4%).

b Proportions are suppressed due to low counts (*n*<6) or reported as ranges to protect privacy.

**Table 2B:** Proportion (row % unless otherwise indicated) of persons with a history of immigration to Ontario (1985-2020) and other Ontarians by COVID-19 vaccination status and difference in three-dose vaccination coverage as of March 13, 2022 across sociodemographic, primary care and immigration characteristics.

		Persons with a history of immigration (row %)				Other Ontarians (row %)				Immigrants vs. other Ontarians
		% 3 doses	% 2 doses	% diff	% 0 doses	% 3 doses	% 2 doses	% 1 dose	% 0 doses	Difference in % 3 doses^a^
	Total Population	46.1	36.0	1.6	16.3	58.2	26.5	1.5	13.8	−12.1
Sex	Female	47.4	35.2	1.6	15.8	61.7	24.4	1.4	12.6	−14.3
	Male	44.7	37.0	1.7	16.7	54.6	28.6	1.7	15.0	−9.9
Age	18-44	39.0	42.0	1.9	17.1	42.1	37.1	2.1	18.7	−3.1
	45-64	51.5	32.6	1.3	14.6	64.2	22.8	1.2	11.8	−12.7
	65+	56.7	23.7	1.7	17.9	79.5	11.9	1.1	7.6	−22.8
Neighbor-hood Income	1 (lowest)	41.3	38.8	2.0	17.9	49.8	29.9	2.3	18.0	−8.5
	2,3,4	46.9	36.1	1.5	15.5	58.0	27.0	1.5	13.6	−11.1
	5 (highest)	51.1	30.9	1.4	16.7	65.9	22.2	1.1	10.8	−14.8
Primary Care	Primary Care Enrollment model	49.0	35.6	1.5	13.9	61.6	25.3	1.4	11.7	−12.6
	Other	40.7	41.5	2.1	15.7	47.9	32.8	2.3	17.0	−7.2
	No primary care	24.8	31.7	1.9	41.5	32.8	31.4	2.3	33.4	−8.0
Neighbor-hood SARS-CoV-2 Risk Deciles	1(highest risk)	41.7	42.4	1.7	14.1	45.9	36.2	1.8	16.1	−4.2
	2	48.2	35.8	1.5	14.6	55.5	29.0	1.5	14.0	−7.3
	3	45.1	36.3	1.7	17.0	56.6	27.7	1.5	14.2	−11.5
	4	44.1	36.5	1.8	17.7	56.5	27.2	1.6	14.6	−12.4
	5	48.5	33.3	1.5	16.7	59.0	25.9	1.5	13.6	−10.5
	6	47.2	33.3	1.6	17.9	59.6	25.4	1.5	13.6	−12.4
	7	49.0	31.4	1.5	18.1	62.1	23.6	1.3	13.0	−13.1
	8	49.7	31.6	1.4	17.3	61.9	23.9	1.3	12.8	−12.2
	9	47.3	32.1	1.5	19.1	60.0	25.3	1.6	13.1	−12.7
	10 (lowest risk)	51.5	28.3	1.5	18.7	59.3	24.9	1.8	14.0	−7.8
Immigration Category	Sponsored Family	43.5	37.4	1.8	17.4					−14.7
	Economic Caregiver	61.5	31.8	0.8	5.8					3.3
	Economic Other	51.7	31.3	1.2	15.8					−6.5
	Resettled Refugee	31.4	47.0	2.8	18.8					−26.8
	Protected persons	35.3	45.9	2.3	16.5					−22.9
	Other immigrants	41.2	39.5	2.1	17.2					−17.0
Region of Birth	Central Africa	17.4	52.7	4.0	26.0					−40.8
	Western Africa	32.4	48.4	2.2	17.0					−25.8
	East Africa	31.7	47.7	2.9	17.7					−26.5
	Southern Africa	61.1	23.1	1.1	14.7					2.9
	Middle East	37.9	42.4	2.2	17.5					−20.3
	North Africa	36.4	41.9	2.0	19.7					−21.8
	Central America	43.6	37.2	1.6	17.6					−14.6
	South America	48.9	35.5	1.5	14.0					−9.3
	Caribbean	30.8	42.2	2.4	24.7					−27.4
	North America	47.9	25.9	1.6	24.5					−10.3
	East Asia	57.9	25.5	1.2	15.4					−0.3
	Australasia & Oceania	52-56^b^	19-22^b^	0-5^b^	20-24^b^					−2.2 to −6.2^b^
	Southeast Asia	65.6	26.4	0.8	7.2					7.4
	South Asia	47.8	40.3	1.4	10.5					−10.4
	Eastern Europe	27.5	38.4	2.1	32.0					−30.7
	Other Europe	44.0	33.0	1.7	21.2					−14.2
	Not Stated	^b^	^b^	^b^	^b^					^b^
Duration of Residence	Under 5 years	40.2	43.7	2.0	14.1					−18.0
	5 to 9 years	41.7	38.0	1.7	18.6					−16.5
	10 to 19 years	45.4	36.6	1.5	16.5					−12.8
	20+ years	50.7	31.7	1.5	16.1					−7.5

a Difference in % for immigration category, region of birth and duration of residence are vs. % 3 doses for other Ontarians overall (58.2%).

b Proportions are suppressed due to low counts (*n*<6) or reported as ranges to protect privacy.

Among immigration characteristics (where each stratum is compared to “other Ontarians” as a whole), region of birth had the largest differences in two and three-dose coverage. Two- and three-dose coverage was lower for all immigration categories except for other economic immigrants, who had similar two-dose coverage, and for economic caregivers who had higher coverage for both two- and three-doses. Two and three dose coverage improved with increasing duration of residence, with much larger differences in coverage comparing immigrants and other Ontarians in three-dose than two-dose coverage, and with these differences becoming smaller with increasing duration.

### All Ontarians: modeling associations with two and three-dose coverage

**Figure 1:**
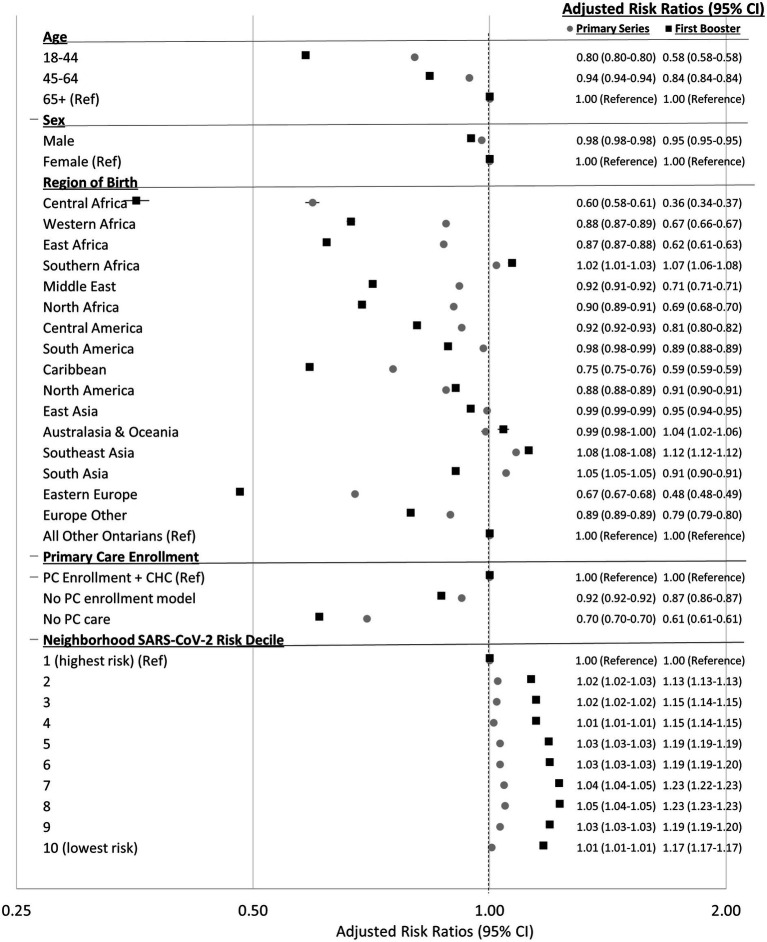
Adjusted risk ratios (95% CI) for receiving two-doses of a COVID-19 vaccine (as of September 13, 2021) and three-doses of a COVID-19 vaccine (as of March 13, 2022) by characteristics of all adult (18+) residents in Ontario, Canada. Risk ratios adjusted for all variables present in figure.

Ontarians with no primary care visits in the previous 2 years and those not enrolled in a primary care model were less likely to receive two doses (aRR = 0.70 [95% CI 0.70–0.70] and aRR = 0.92 [95% CI 0.92–0.92], respectively) and three doses (aRR = 0.61 [95% CI 0.61–0.61] and aRR = 0.86 [95% CI 0.86–0.87], respectively) compared to those enrolled in a primary care model (Figure 1). Immigrants from most regions of birth were less likely to receive two doses, and even less likely to receive three doses, compared to other Ontarians. Central Africans were the least likely to receive two (aRR = 0.60 [95% CI 0.58–0.61]) and three doses (aRR = 0.36 [95% CI 0.34–0.37]), followed by Eastern Europeans (aRR = 0.67 [95% CI 0.67–0.68] and aRR = 0.48 [95% CI 0.48–0.49], respectively) and Caribbeans (aRR = 0.75 [95% CI 0.75–0.76] and aRR = 0.59 [95% CI 0.59–0.59], respectively). South Africans and Southeast Asians were slightly more likely to receive two (2 and 8%, respectively) and three doses (7 and 12%, respectively). South Asians were more likely to receive two (aRR = 1.05 [95% CI 1.05–1.05]) but less likely to receive three doses (aRR = 0.91 [95% CI 0.90–0.91]). Two-dose and three-dose coverage was the most similar among those from South Africa, North America, Australasia & Oceania, Southeast Asia, and East Asia with the former four regions having slightly greater three- than two-dose coverage.

### Persons with a history of immigration: modeling associations with two and three-dose coverage

**Figure 2:**
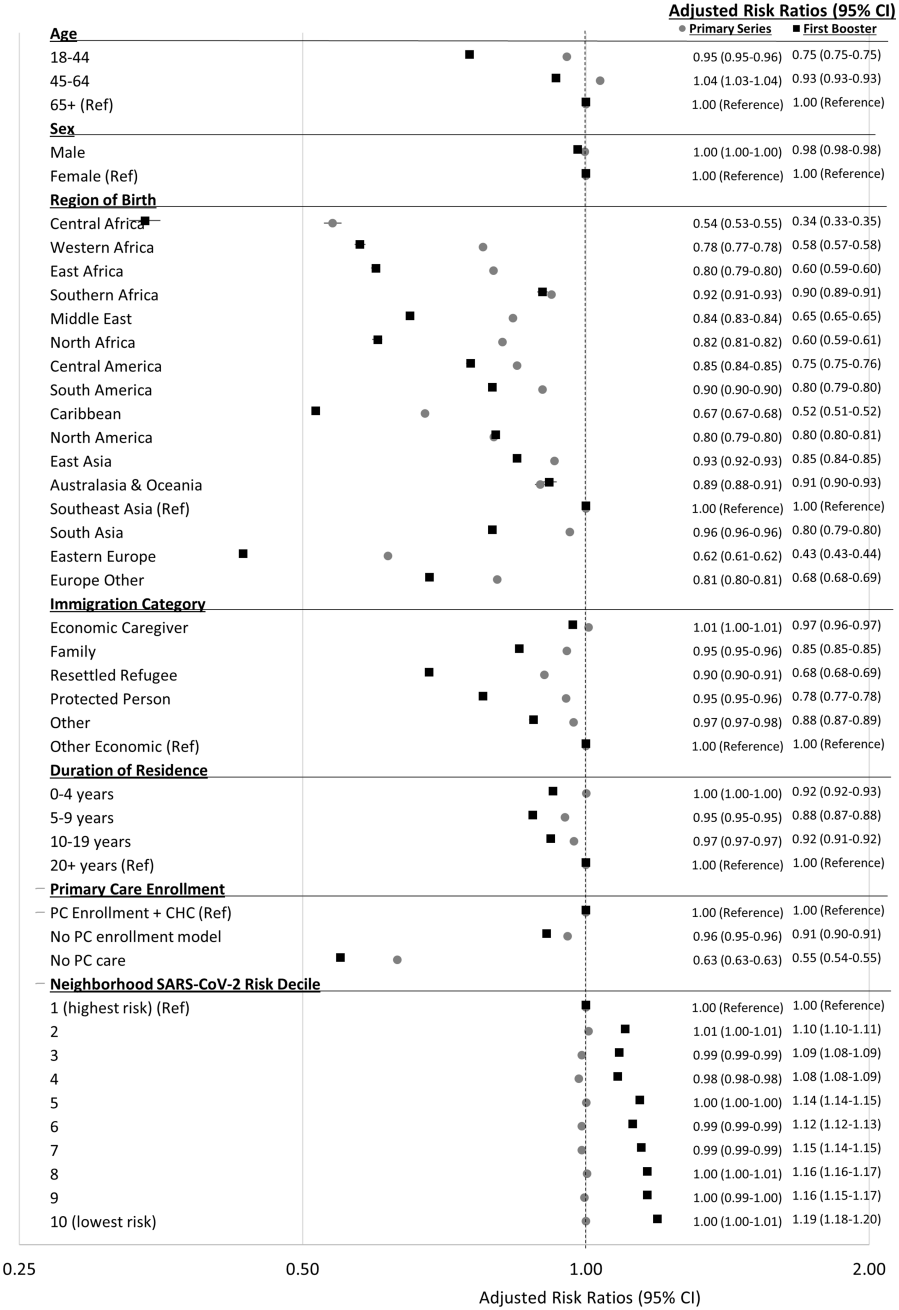
Adjusted risk ratios (95% CI) for receiving two-doses of a COVID-19 vaccine (as of September 13, 2021) and three-doses of a COVID-19 vaccine (as of March 13, 2022) by characteristics of adults (18+) with a history of immigration in Ontario, Canada. Risk ratios adjusted for all variables present in figure.

Similar relative trends to the “all Ontarians” model described above were seen for these immigrant region of birth analyses, except here comparisons are to Southeast Asians (Figure 2). Compared to other economic immigrants, economic caregivers were just as likely to receive two doses, and slightly less likely to receive three doses. Resettled refugees, protected persons, sponsored family, and other immigrants were 3–10% less likely to receive two doses and 12–32% less likely to receive three doses compared to other economic immigrants. Compared to immigrants residing in Canada for 20+ years, those in Canada for 0–4, 5–9, and 10–19 years were equally likely or up to 12% less likely to receive two or three doses.

## Discussion

In this retrospective population-based study in Ontario, Canada we found that across most strata of socio-demographic, primary care and immigration characteristics, two-dose COVID-19 vaccine coverage was reasonably equitable, with similar or slightly lower coverage among immigrants compared to other Ontarians. Immigrants residing in the higher SARS-CoV-2 risk deciles had similar two-dose coverage compared to other Ontarians and immigrants residing in all but the highest neighborhood income quintile had similar two-dose coverage as other Ontarians. These findings contrast with three-dose coverage, where across almost all strata coverage was much lower among immigrants compared to other Ontarians. All Ontarians, and immigrants especially, with no primary care visits in the last 2 years were less likely to receive two or three-doses compared to those enrolled in a primary care model. Associations of immigrant region of birth with two and three-dose coverage indicated highly variable coverage, with much lower coverage of both two and three doses among Central Africans, Eastern Europeans, Caribbeans, Western and East Africans, Middle Easterners and North Africans, and slightly higher coverage among those from Southeast Asia and South Africa compared to other Ontarians. Of all immigration categories, resettled refugees and protected persons were the least likely to receive three-doses (vs. economic immigrants). We observed weak adjusted associations between recency of immigration and two and three-dose coverage.

Studies from Norway (5), Italy (6), and Finland (8) found that im/migrants were less likely to be vaccinated compared with their counterparts born in the country. The Norwegian study (5) found that immigrants born in some Eastern European countries had higher odds of not having at least one dose of a COVID-19 vaccine, while those born in Southeast and South Asian countries had slightly lower or equal odds. Similarly, Finnish migrants from Estonia, Africa (excluding North Africa) and Russia/Former Soviet Union had lower odds of not having complete vaccine coverage (i.e., SARS-CoV-2 infection + one COVID-19 vaccine dose, or two COVID-19 vaccine doses) whereas migrants from Southeast Asia, Asia and Middle East/North Africa had greater odds. A study from the province of Alberta (Canada) (7) reported that 2% more immigrants (78.2%) than non-immigrants (76.0%) received at least one COVID-19 vaccine dose, however these, nor the results by region of birth, were adjusted for confounding and immigration category was not examined, making direct comparison to our study difficult. A study from Italy (9) and a study abstract from the United Kingdom (10) found among those with at least one COVID-19 vaccine, migrants took longer to receive their first dose (9, 10) particularly those from Africa, Europe, and the Eastern-Mediterranean (9) and in the receipt of the second and third doses among refugees (10). A recent systematic review examining determinants of vaccination in migrant populations in Europe (4) (which included no studies examining determinants of COVID-19 vaccination) found geographic origin (African, Eastern, or Central European origin and Eastern Mediterranean or Middle Eastern origin), recent migration, being a refugee or asylum-seeker and not having accessed healthcare in more than a year to be the most associated with under-vaccination.

We found immigrants were either equally or only slightly less likely than other Ontarians to receive two doses across most strata, suggesting that during the promotion of the primary series (prior to starting the mandatory vaccine program), government policies promoting equitable distribution, public health outreach, and community-led strategies (23–25) were largely successful in achieving equity for immigrants and refugees in Ontario. This seems particularly evident in the two highest risk SARS-CoV-2 risk deciles and four of five neighborhood income quintiles where there was no difference in two-dose coverage between immigrants and other Ontarians. The wider inequities in three-dose coverage within strata and lower aRRs have important implications. Without up-to-date COVID-9 vaccination, under-immunized and unimmunized populations are at increased risk of infection and severe outcomes, particularly with the removal of all public health protections and sustained levels of SARS-CoV-2 in Ontario since June 2022 (33). These findings suggest an urgent need to return to the policies and programs which encouraged and successfully removed barriers and facilitated access to two-doses, with a continued commitment to reduce inequities.

Generally, barriers to vaccination among migrants are categorized by access, affordability, awareness, acceptance, and activation (4). Access barriers are the most prominent and include language/literacy barriers, resource and capacity constraints, practical and provider-level barriers and distrust of the health system and authorities. Acceptance barriers include cultural and social barriers, worries about safety, historic and current adverse community-level experiences, misinformation or lack of information, and low perception of risk. Awareness barriers include low health literacy, the need for vaccines or boosters and where to access vaccines. Affordability barriers include costs and competing priorities. Finally, activation barriers are lack of information or practical support from health-care professionals and blanket approaches. Regarding COVID-19 vaccination, distrust in the healthcare system and government and vaccine safety concerns were cited among migrants and ethnic minority groups globally (3) and in a recent Canadian survey (34). For ongoing COVID-19 vaccination, the relevance of these barriers may vary with time and dose—e.g., worries about side-effects may be relevant for the first dose whereas access barriers may fluctuate over time.

The wide variability in vaccine coverage by immigrant region of birth suggest that the barriers and facilitators to vaccination are highly heterogeneous across the diverse group of immigrants in Ontario. Southeast Asian and South Asian immigrants appear well-served by vaccine policies and outreach. Many Southeast Asians were likely employed in the healthcare sector and other frontline essential sectors (35), who were prioritized for earlier and targeted vaccine distribution. The South Asian community, particularly in one major region in the Greater Toronto Area (Peel), was extremely hard hit by SARS-CoV-2 (25, 36). Eventually Peel received an adequate vaccine supply, and held mass vaccination and pop-up clinics with linguistically and culturally appropriate outreach (37).

The necessity for directly involving im/migrant communities in COVID-19 vaccination efforts has been acknowledged repeatedly (3, 4, 11, 13, 38). These efforts establish and build trusted and meaningful relationships, facilitate understanding, and overcome barriers to vaccination in culturally humble and structurally competent ways. In Toronto, where ~70% of Ontario’s immigrant population resides, the regional health authority’s Vaccine Ambassador program increased confidence, access, and uptake using an innovative community-centered model (24). In one of many community-driven initiatives in Toronto’s Black communities, the Black Scientists’ Task Force on Vaccine Equity (23) substantially increased vaccination among healthcare staff and reduced community-level vaccine hesitancy. This initiative addressed distrust of health care providers and the healthcare system (due to historical and contemporary experiences of racism in Canada and countries of origin), misinformation and logistical difficulties accessing vaccines (39, 40). To reach immigrants who are more likely to self-identify as Black who remain unvaccinated or under-vaccinated (i.e., Central, West, East African, and the Caribbean), it may be worth re-invigorating these efforts and expand into neighborhoods where these events have not yet occurred.

In our study, Eastern European immigrants were among those with the lowest coverage. This is consistent with findings from Eastern Europe where vaccine confidence is the lowest of any sub-region in the world (41). In the United Kingdom, low vaccination in the Polish diaspora was linked to exposure to Polish media and social media where there is a thriving anti-vaccine movement (13). After authors of this study provided Toronto’s public health authority with similar analyses, outreach to the Eastern European diaspora was conducted to improve vaccination.

Prior to the availability of COVID-19 vaccines, guidance was emerging concerned with ensuring im/migrant populations’ equitable access to COVID-19 vaccines (42). This guidance described the need for mobile and mass vaccination clinics, delivery of inclusive health services via cultural mediators and interpreters and a culturally competent healthcare service, culturally and linguistically appropriate immunization messaging, and improving migrants’ awareness of their health rights (42). Others have since also highlighted the positive impact community leaders and trusted individuals disseminating accurate and appropriate vaccine information had on vaccine uptake (43). In addition, based on a rapid review of evidence, in March 2022 the World Health Organization released a guide describing priority areas for action (44). The guide outlined six domains including: using and collecting social and behavioral data to drive decision-making, coordination of inclusive policy and planning, implementing communication strategies, social media monitoring and misinformation management, people-centered and community-led approaches, and capacity-building and training.

Our findings are not generalizable to temporary residents or undocumented immigrants since neither group can be identified by the immigration dataset. Despite efforts to link vaccine records without a health card number using other identifiers, unlinked records were excluded, and it is unknown whether and how these systematically differ from the population. We could not account for SARS-CoV-2 infection (due to poor reporting of test results after Nov 2021), which would delay eligibility for the third dose. While we conceptualized “other Ontarians” as having the fewest barriers to vaccination, we acknowledge there are subgroups who face barriers. The federal immigration dataset used in this study does not identify immigrants who originally arrived in another Canadian province and re-migrated to Ontario and therefore they could not be separately examined; this small group of immigrants are therefore included in the “other Ontarians” reference group. Our study has several strengths. It is only one of six studies examining COVID-19 vaccination among immigrants in the literature. Among these studies, ours is population-based with a very large (~2.5 million) and diverse group of immigrants, and the first to report on three-dose coverage. We identified wide inequities in COVID-19 vaccination by highly disaggregated regions of birth which may relate to intervenable structural, cultural, linguistic, and political barriers.

In conclusion, despite reasonably equitable two-dose coverage among immigrants compared to other Ontarians across many characteristics, equitable vaccination was not sustained for the third dose. With the removal of all public health protections in April 2022, cases of SARS-CoV-2 have risen and remain steady across Ontario. Those that remain unvaccinated or under-vaccinated are at higher risk of COVID-19 related morbidity and mortality, suggesting a need to re-invigorate public health policies and practices that minimized inequities and facilitated high two-dose coverage. Given the wide-variability and mostly lower uptake by immigrant region of birth, there is a need to cultivate community participatory engagement strategies to overcome barriers and encourage uptake of the bivalent COVID-19 vaccine. Our findings are valuable for ongoing COVID-19 vaccination programs and public health strategies in Canada and other high-income countries.

## Data availability statement

The data analyzed in this study are subject to the following licenses/restrictions: the data sets from this study are held securely in coded form at ICES. Data-sharing agreements prohibit ICES from making the data sets publicly available, but access may be granted to those who meet pre-specified criteria for confidential access, available at https://www.ices.on.ca/DAS. The data set creation plan and the underlying analytic code are available from the authors upon request, understanding that the programs may rely upon coding templates or macros that are unique to ICES. Requests to access these datasets should be directed to https://www.ices.on.ca/DAS.

## Ethics statement

Ethical approval was not required for the study involving humans in accordance with the local legislation and institutional requirements. Written informed consent to participate in this study was not required from the participants or the participants’ legal guardians/next of kin in accordance with the national legislation and the institutional requirements. ICES is a prescribed entity under Ontario’s Personal Health Information Protection Act (PHIPA). Section 45 of PHIPA authorizes ICES to collect personal health information, without consent, for the purpose of analysis or compiling statistical information with respect to the management of, evaluation or monitoring of, the allocation of resources to or planning for all or part of the health system. Projects that use data collected by ICES under section 45 of PHIPA, and use no other data, are exempt from REB review. The use of the data in this project is authorized under section 45 and approved by ICES’ Privacy and Legal Office.

## Author contributions

SW, AG, SG, and HL conceived of and designed the study and along with TS developed the methodological approach. JB, JS, and SW conducted the literature search and synthesized the existing literature. HL conducted formal analysis and SW developed the figures and data visualization. SW and AG wrote the original draft. SW, HL, SG, JS, TS, P-PP-R, JB, SA, and AG contributed to the interpretation of the data. AG acquired funding. All authors contributed to the article and approved the submitted version.

## Funding

Supported by ICES (formerly the Institute for Clinical Evaluative Sciences) and funded by the Canadian Institutes for Health Research (grant PJT-155917, principal applicant Guttmann). The funders had no role in the writing of the manuscript or the decision to submit the manuscript for publication. No author has been paid to write this article by a pharmaceutical company or other agency. The authors were not precluded from accessing data in the study and accept responsibility for publication.

## Conflict of interest

P-PP-R has been a co-investigator on an investigator-led project funded by Pfizer that is unrelated to this study.

The remaining authors declare that the research was conducted in the absence of any commercial or financial relationships that could be construed as a potential conflict of interest.

## Publisher’s note

All claims expressed in this article are solely those of the authors and do not necessarily represent those of their affiliated organizations, or those of the publisher, the editors and the reviewers. Any product that may be evaluated in this article, or claim that may be made by its manufacturer, is not guaranteed or endorsed by the publisher.
