# Supplementary material for: COVID-19 vaccine equity: a retrospective population-based cohort study examining primary series and first booster coverage among persons with a history of immigration and other residents of Ontario, Canada
Source: Front Public Health. 2023 Sep 8;11:1232507. doi: 10.3389/fpubh.2023.1232507 (PMC10515385; doi:10.3389/fpubh.2023.1232507)

**Supplementary Material**

**Wanigaratne et al, 2023 - COVID-19 vaccine equity: a retrospective population-based cohort study examining primary series and first booster coverage among persons with a history of immigration and other residents of Ontario, Canada.**

**Supplementary Table 1: Description of linked administrative health and demographic databases, corresponding variables used in current study.**

| **Name of Database** | **Data Provider** | **Description** |
| --- | --- | --- |
| Client Agency Program Enrolment (CAPE) | Ontario Ministry of Health | Indicates the enrollment of an individual in a primary care program with a specific practitioner or group. |
| Community Health Centres (CHCs) | Alliance for Healthier Communities | CHCs deliver primary care services in combination with health promotion and illness prevention services, primarily in high need areas to individuals who do not have a health care provider, are newcomers to Canada, face barriers to care, have mental health or addiction issues, require counseling or help with housing issues, and/or have no health insurance. Care is provided by physicians, nurse practitioners, nurses, counsellors, community workers, and dietitians. The dataset includes chart abstracted records for all visits in all such centres across Ontario. The dataset is compiled and validated by the Alliance for Healthier Communities before being sent to ICES for use in healthcare research. Data is updated annually and records are linked using individuals encrypted health card numbers. None of the care provided by CHCs is captured in OHIP and thus CHC data represents a separate source of primary care access, particularly by vulnerable Ontarians. |
| Corporate Provider Database (CPDB) | Ontario Ministry of Health | Contains information on providers (e.g., physicians, nurses) and groups (e.g., primary care, hospitals) eligible to receive payment from OHIP. |
| Immigration, Refugees, and Citizenship Canada’s Permanent Resident Database | Immigration & Refugees and Citizenship Canada (IRCC) | Permanent residents’ demographic information collected by the IRCC during the immigration application process. Overall 86.4% of immigrants and refugees in the IRCC database were linked to the healthcare registry, with minimal variability by immigration category, region of birth and minimal differences in characteristics between linked and unlinked refugees. (Chiu et al, 2016) |
| OHIP Registered Persons Database (RPDB) (healthcare registry) | Ontario Ministry of Health | Basic demographic information (age, sex, location of residence, date of birth, and date of death for deceased individuals) for all individuals issued an Ontario health insurance number. Also indicates the time periods individuals are eligible for health insurance and provides the best-known postal code for each registrant on July 1st of each year which is updated yearly. Linkable to all other ICES data holdings using an encrypted health card number. |
| Ontario COVID-19 vaccine data (COVaxON) | Ontario Ministry of Health  Public Health Ontario (PHO) | The central provincial database and point-of-care system supporting COVID-19 vaccination. The Ministry of Health compiles and manages these individual level data and Public Health Ontario provide ICES with a client-level dataset detailing vaccination records. While vaccination records have been cleaned using data cleaning logic, in instances where multiple records remain present for an individual, the records are reconciled manually based on the most likely scenario (1. 2^nd^ record represents a second vaccination event [ie. 2^nd^ dose]; 2. Repeat record; 3. Incorrectly entered identifier (in which case the earliest record was selected) |
| Ontario Health Insurance Plan (OHIP) | Ontario Ministry of Health | Contains information on inpatient and outpatient services provided to Ontario residents eligible for health insurance by fee-for-service health care practitioners (primarily physicians) and “shadow billings” for those paid through non-fee-for-service payment plans. Billing codes on the claims (OHIP fee codes) identify the care provider, their area of specialization and the type and location of service. OHIP billing claims also contain a 3-digit diagnosis code – the main reason for the service – captured using a modified version of the ICD, 8^th^ revision coding system. |
| Ontario Laboratories Information System (OLIS) | Ministry of Health  eHealth Ontario | Contains lab orders, test requests and lab results from most laboratories in Ontario. Starting April 7 2020, ICES began receiving daily cumulative updates of COVID-19 test orders from eHealth Ontario (eHO, now part of Ontario Health Digital Health Services). These data are a minimum dataset extracted from lab orders with COVID-19-specific test request (TR) or LOINC codes and other TR/LOINC codes indicative of viral or respiratory virus testing. Each record represents a testing event, with testing events linkable by encrypted individual health card numbers. |
| Postal Code Conversion File (PCCF)+ | Statistics Canada | A conversion template between the six-character postal code and Statistics Canada's standard geographic areas. Through the link between postal codes and standard geographic areas, the PCCF permits the integration of data from various census derived . |
| Public Health Case and Contact Management (CCM) Solutions | Public Health Ontario (PHO) | A central data repository for COVID-19 case and contact management, and reporting in Ontario. The Health Protection and Promotion Act requires that each public health unit in Ontario collect information about people with diseases of public health significance (reportable diseases), including COVID-19, in their jurisdiction and report it to the Ministry of Health (MOH). This information is used for local, provincial and national surveillance. |

**Supplementary Table 2: List of Variables**

| **Variables Used in this study** | **Database Source** | **Variable Definition & Categories** | **Collected/**  **measured at** |
| --- | --- | --- | --- |
| Age category | OHIP Registered Persons Database (RPDB) (healthcare registry) | Calculated using the index date (Jan 1, 2021) and the date of birth recorded in the RPDB. Categorized into: 18-44 years, 45-64 years and 65+ years. | At study index time (Jan 1, 2021) |
| Sex | OHIP Registered Persons Database (RPDB) (healthcare registry) | Sex (male/female) of the individual as recorded in the individual’s health card. We recognize that this binary categorization does not necessarily reflect gender identity. | Health care registration/renewal |
| Other Ontarians (i.e., those not found in the IRCC database) | OHIP Registered Persons Database (RPDB) (healthcare registry) | This group includes: i) those born in Ontario (the majority), ii) interprovincial migrants who were either immigrants to Canada or born in Canada but who moved to Ontario between 1991 and 2020, and iii) immigrants to Ontario who are permanent or temporary residents who arrived in Ontario after September 30, 2020. | Health care registration/renewal |
| Immigration category | Immigration, Refugees, and Citizenship Canada’s (IRCC) Permanent Resident Database | - Resettled refugees: government sponsored refugees, privately sponsored refugees, blended visa office referred refugees. - Protected persons: successful asylum-seekers. - Sponsored Family: sponsored by a Canadian family member - Economic Caregivers: predominately females born in the Philippines who entered Canada as temporary migrant workers employed as caregivers for children or persons with medical needs; after becoming permanent residents many remain employed in the caregiving sector and other essential work sectors, an important risk factor for SARS-CoV-2 exposure. - Other Economic Immigrants: must meet criteria for education, official language ability and employment skills along with few pre-existing medical conditions. - Other Immigrants: humanitarian & compassionate, designated backlog clearance, deferred removal order, permit holder class, express entry | Time of immigration application |
| Country of birth | Immigration, Refugees, and Citizenship Canada’s (IRCC) Permanent Resident Database | Countries categorized into 17 regions using the United Nations M49 Standard Classification Methodology - Central Africa, Western Africa, East Africa, Southern Africa, Middle East, North Africa, Central America, South America, Caribbean, North America, East Asia, Australasia & Oceania, Southeast Asia, South Asia, Eastern Europe, Other Europe, Not Stated. | Time of immigration application |
| Duration of residence | Immigration, Refugees, and Citizenship Canada’s (IRCC) Permanent Resident Database | Calculated using the index date (Jan 1, 2021) and the date of arrival recorded in the IRCC database. Categorized into: 0-4 years, 5-9 years, 10-19 years, 20+ years | Index date (Jan 1, 2021) |
| Neighborhood income quintile (based on postal code in health care registry) | Postal Code Conversion File (PCCF)+  OHIP Registered Persons Database (RPDB) (healthcare registry) | Based on the residential postal code recorded in the RPDB as of July 1, 2022 and the Statistics Canada census of 2016.  Categorized into Q1 [lowest neighborhood income], Q2, Q3, Q4 and Q5 [highest neighborhood income]. Those areas with suppressed income data were merged with the lowest income, as they are usually low income. | 2016 census |
| Primary Care Enrollment | Client Agency Program Enrolment (CAPE)  Corporate Provider Database (CPDB)  CHC  Ontario Health Insurance Plan (OHIP) | Primary Care Enrollment model categorized into: i) *Primary care enrollment model* - includes patients enrolled in a Family Health Group (FHG), Family Health Network (FHN), Family Health Organization (FHO), or who visited a Community Health Centre (CHC), ii) *Not enrolled in a primary care model* – those not enrolled in a primary care model were assigned to the primary physician with the highest dollar value of all billings for primary care visits; includes general practitioners and family physicians practicing outside of primary care models (not providing comprehensive services) and work in walk-in clinics or solo-practices, and iii) *No primary care visits in last 2 years* – those with no primary care affiliation and no primary care claims in the previous 2 years. | Index date – Jan 1, 2021 |
| Vaccination status with any Health Canada approved COVID-19 vaccine:  a) Pfizer,  b) Moderna  c) AstraZeneca/COVISHIELD | Ontario COVID-19 vaccine data (COVaxON) | 0, 1 and 2 doses as of Sept 13, 2021  0, 1, 2 and 3 doses as of March 13, 2022 | Dec 2020 to March 13, 2022 |
| Neighborhood SARS-CoV-2 Risk Deciles | Ontario Laboratories Information System (OLIS)  Public Health Case and Contact Management (CCM) Solutions | Cumulative incidence of SARS-CoV-2 cases for each Forward Sortation Area (FSA; consisting of an average of 8000 households) from individuals living outside of long-term care facilities. FSAs were ranked by their cumulative incidence and grouped into deciles so that each decile had 10% of the population. Each decile represents the neighborhood risk of SARS-CoV-2, where 1 = highest incidence neighborhoods and 10 = lowest incidence neighborhoods.  In April 2021, the Ontario government allocated additional vaccines to 30% (n=114) of neighborhoods which had historically high rates of COVID-19 related deaths and hospitalizations which aligned very closely with the highest of these neighborhood risk deciles; 50% of available vaccines were allocated to these neighborhoods for two weeks in May 2021 with the age of eligibility lower than in other neighborhoods. | As of March 28, 2021 |

**Supplementary Table 3: Ontario’s COVID-19 vaccine rollout: important dates and context**

| **Dose #** | **Rolling eligibility dates and details** | **Provincial context** |
| --- | --- | --- |
| First dose | December 14 2020 to May 30, 2021 | - Oct 2020 - Delta strain emerges in Ontario. - Dec 14, 2020 to April 8, 2021 - initial eligibility based on per-capita regional allocation with subsequent distribution by age, chronic health conditions and high-risk congregate settings, SARS-CoV-2 hotspots and essential worker status - Ontario government allocates additional vaccines to “hotspot” neighborhoods[14] - April 27 to May 23, 2021 – gradual dropping of age eligibility to 12+ with simultaneously lower age eligibility in “hotspot” communities - During the weeks of May 3^rd^ and May 10^th,^ 50% of provincial vaccine supply allocated to “hotspot” neighborhoods |
| Second dose | May 31, 2021 to September 2021 | - Gradual eligibility based on descending age, timing of first dose and residence in “hotspots” - June 2021 Delta becomes dominant strain in Ontario. - July 2021 ON govt provides data on vaccination status of rostered patients to primary care physicians in enrollment models. While physician offices were not the venue for vaccination, some physicians conducted home visits or organized outdoor clinics. - September 22, 2021 proof of 2-dose vaccination implemented for high-risk settings |
| Third dose | September 2021 to December 2021 | - Sept 2021 - select vulnerable populations eligible for 3^rd^ dose - Oct 2021 ICES provides public health units with large immigrant populations with disaggregated data analyses outlining under-vaccinated groups - Nov 2021 Omicron variant first detected. - 3^rd^ dose eligibility: Dec 13 – 50+ eligible; Dec 22 – 18+ eligible |
| Fourth dose | January 2022 to July 14, 2022 | - March 2022 - Omicron variant becomes dominant strain in Ontario. - March 1, 2022 – mandatory vaccination program ends - March 21, 2022 – removal of mask mandates in childcare settings, schools and most workplaces. - June 11, 2022 – removal of mask mandates for employees and the public on transit and in health-care settings other than long-term care and retirement homes. |

**Supplementary Figure 1 – Cohort diagram**


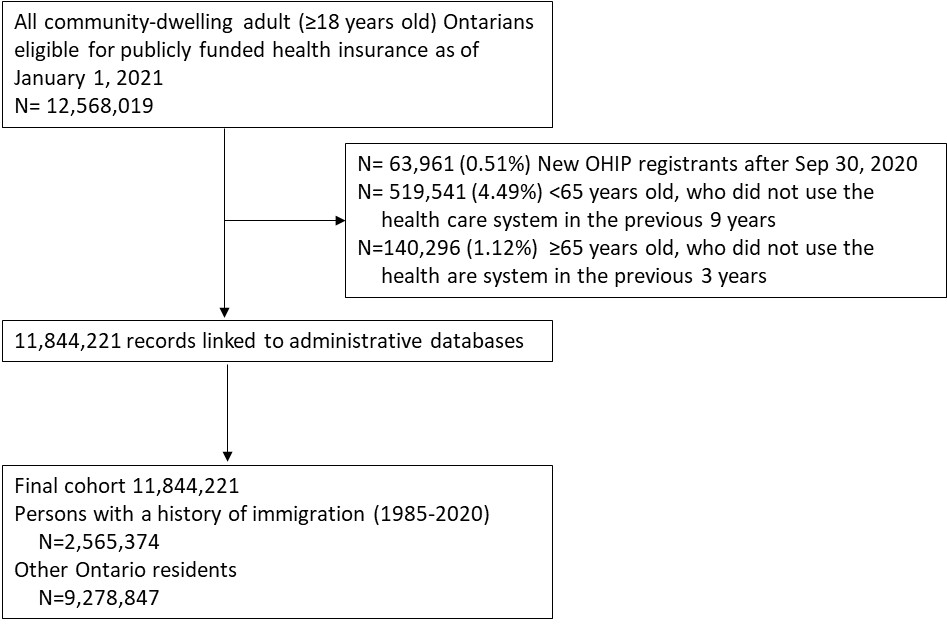

Supplement: Supplementary file 1 [file Table_1.docx]
